# Properties of Dislocation Drag from Phonon Wind at Ambient Conditions

**DOI:** 10.3390/ma12060948

**Published:** 2019-03-21

**Authors:** Daniel N. Blaschke

**Affiliations:** Los Alamos National Laboratory, Computational Physics Division, Los Alamos, NM 87545, USA; dblaschke@lanl.gov

**Keywords:** dislocations in crystals, drag coefficient, phonon wind

## Abstract

It is well known that, under plastic deformation, dislocations are not only created but also move through the crystal, and their mobility is impeded by their interaction with the crystal structure. At high stress and temperature, this “drag” is dominated by phonon wind, i.e., phonons scattering off dislocations. Employing the semi-isotropic approach discussed in detail in a previous paper (*J. Phys. Chem. Solids* **2019**, *124*, 24–35), we discuss here the approximate functional dependence of dislocation drag *B* on dislocation velocity in various regimes between a few percent of transverse sound speed $c_{T}$ and $c_{T}$ (where $c_{T}$ is the effective average transverse sound speed of the polycrystal). In doing so, we find an effective functional form for dislocation drag B(v) for different slip systems and dislocation characters at fixed (room) temperature and low pressure.

## 1. Introduction

Many modern material strength models, for example those in Refs. [1,2,3,4,5,6,7], are based on dislocation dynamics. However, dislocation mobility, especially in the high temperature and high stress regime, is poorly understood theoretically. Moving dislocations experience a drag due to their interaction with the crystal structure, and this drag coefficient *B* determines the dislocation glide time between obstacles. The lack of a well-established functional form for B(v,T,…) has led many researchers to assume *B* to be a constant (or a constant over a simple “relativistic” factor) as a fist order approximation within their strength models. Thus, better insight into the true functional form of *B* could improve those models.

Different mechanisms dominate dislocation drag in different regimes. However, at temperatures comparable to or higher than the Debye temperature and at high stress (leading to dislocation velocities in the range $0.01≲v/c_{S}<1$, where $c_{S}$ denotes the lowest shear wave speed corresponding to the direction of dislocation glide), phonons scattering off dislocations (commonly referred to as “phonon wind”) constitutes the dominating effect. The lower end of this range is known as the “viscous” regime where B(v) at given temperature and pressure is known to be roughly constant. However, with increasing stress and thus increasing dislocation velocity, *B* exhibits a non-linear velocity dependence. This is seen in numerous molecular dynamics (MD) simulations (see e.g., [8,9,10,11,12] and references therein), but also within the recent theoretical framework in Refs. [13,14].

In Ref. [13], the theory developed by Alshits and collaborators [15] is taken to the next level by including not only the full velocity dependence of *B*, but also longitudinal phonons (in addition to the dominating contribution of transverse phonons) as well as an anisotropic dislocation field and single crystal elastic constants. Hence, this model developed having polycrystals in mind, keeps the phonon spectrum isotropic (for simplicity), but dislocations are modeled according to the single crystal symmetry (bcc, fcc, hcp, etc.) in order to take into account their anisotropy to some extent. This “semi-isotropic” approach constitutes an intermediate step in an ongoing long-term endeavor to include all anisotropic effects and the true phonon spectrum (which is beyond the scope of the current work). Nonetheless, valuable insights were already gained, e.g., the non-trivial dependence of the drag coefficient on the dislocation character angle ϑ (between line sense and Burgers vector).

For now, the model is also restricted to the subsonic regime where $v<c_{T}$. The question whether dislocations in metals can reach supersonic speeds is still under debate, although numerous MD simulations suggest it is possible [8,11,16,17,18,19]; see also the recent discussion on interpreting those results in the context of line tension and dislocation shape [20]. For recent literature on supersonic dislocations, (see, e.g., [21,22,23] and references therein).

Here, our main goal is to highlight the effective functional dependence of *B* on the dislocation velocity within the theory of [13] (and its numerical implementation of Ref. [24]), and to explain how to derive simple analytic representations of B(v) that are amenable to subsequent use in applications (such as material strength models). We also present new results for metals and slip systems not presented in [13] (i.e., prismatic and pyramidal slip for hcp metals). Thus, the current work in a sense complements Ref. [13] and the theory developed there.

## 2. Phonon Wind in the Semi-Isotropic Approach

The drag coefficient *B* of a dislocation is defined as the proportionality coefficient of the force F=Bv needed to maintain dislocation velocity *v*. It is related to the dissipation *D* per unit length due to phonon scattering via $D=Bv^{2}$, and takes the form [25,26]

(1) $$\begin{array}{c} B=\frac{4\pi }{\hbar v^{2}}\sum_{s^{\prime },s^{\prime \prime }}\sum_{q^{\prime },q^{\prime \prime }}\int \;\;d^{2}q\;\Omega_{q}{\left|\Gamma_{s^{\prime }s^{\prime \prime }}\left(\vec{\;q}{\;}^{{\;}^{\prime }},\vec{\;q}{\;}^{{\;}^{\prime \prime }},\vec{\;q}\right)\right|}^{2}\left(n_{q^{\prime \prime }}-n_{q^{\prime }}\right)\delta \left(\vec{\;q}{\;}^{{\;}^{\prime }}-\vec{\;q}{\;}^{{\;}^{\prime \prime }}-\vec{\;q}\right)\delta \left(\omega_{q^{\prime }}-\omega_{q^{\prime \prime }}-\Omega_{q}\right)\;,\\ \Gamma_{s^{\prime }s^{\prime \prime }}\left(\vec{\;q}{\;}^{{\;}^{\prime }},\vec{\;q}{\;}^{{\;}^{\prime \prime }},\vec{\;q}\right)=\frac{\hbar }{4\rho \sqrt{\omega_{q^{\prime }}\omega_{q^{\prime \prime }}}}\sum_{i,j,k}d_{kk^{\prime }}\left(\vec{\;q}\right)w_{q^{\prime }i}^{*}w_{q^{\prime \prime }j}\sum_{i^{\prime }j^{\prime }k^{\prime }}q_{i^{\prime }}^{\prime }q_{j^{\prime }}^{\prime \prime }{\tilde{A}}_{ijk}^{i^{\prime }j^{\prime }k^{\prime }} \end{array}$$

where $\vec{\;q}{\;}^{{\;}^{\prime \prime }}$ and $\vec{\;q}{\;}^{{\;}^{\prime }}$ are the wave vectors of incoming and outgoing phonons, $s^{\prime }$ and $s^{\prime \prime }$ label their polarizations (two transverse and one longitudinal), and $\vec{\;q}$ is the wave vector associated with the dislocation field in Fourier space, $d_{kk^{\prime }}$. In contrast to the phonons (which are quantized and have discrete wave vectors determined from the perfect lattice), the dislocation is modeled as a classical field in the continuum limit. Assuming an infinitely long, straight dislocation, its only spatial dependence is within the plane perpendicular to the dislocation line. The sums over discrete phonon momenta can subsequently be approximated as integrals over the first Brillouin zone. $\omega_{q^{\prime }}$, $\omega_{q^{\prime \prime }}$ are the phonon frequencies presently depending linearly on the wave vector length in accordance with the isotropic Debye approximation, i.e., $\omega_{q^{\prime }}=c_{s^{\prime }}\left|\vec{\;q}{\;}^{{\;}^{\prime }}\right|$ where $c_{s^{\prime }}$ is the sound speed of a phonon with polarization $s^{\prime }$ (either transverse $c_{T}$ or longitudinal $c_{L}$). $\Omega_{q}=\left|\vec{\;q}\cdot \vec{\;v}\right|$ is the energy transfer whenever a phonon scatters on the dislocation. $n_{q^{\prime }}$ denotes the equilibrium phonon distribution function $n_{q^{\prime }}={\left(\exp\left(\hbar \omega_{q^{\prime }}/k_{B}T\right)-1\right)}^{-1}$, which controls the number of scattering events per unit time. *ℏ* is Planck’s constant, ρ is the material density, and the two Dirac delta functions in the second line of Equation (1) encode momentum and energy conservation within each scattering event. Γ finally represents the associated matrix element, or scattering probability. As such, it depends on the (anisotropic) dislocation displacement gradient field $d_{kk^{\prime }}$, the (quantized, isotropic) phonons whose orthonormal polarization vectors are presently denoted by $w_{qi}\;:=w_{i}\left(\vec{\;q},s\right)$, and a linear combination of second (SOEC) and third order elastic constants (TOEC) of the anisotropic single crystal grains of a polycrystal, ${\tilde{A}}_{ijk}^{i^{\prime }j^{\prime }k^{\prime }}$. For technical details on the theory, we refer to [13] as well as [14,15].

### 2.1. Steady State Dislocations and Slip Geometries

The displacement gradient field in the continuum limit and within the realm of linear elasticity of a dislocation moving at constant velocity, can be determined from solving the equations of motion (e.o.m.) and the (leading order) stress–strain relations known as Hooke’s law:

(2) $$\begin{array}{cccc} \partial_{i}\sigma_{ij} & =\rho {\ddot{u}}_{j}\;, & \sigma_{ij} & =C_{ijkl}\epsilon_{kl}=C_{ijkl}u_{k,l}, \end{array}$$

where we have introduced the notation $u_{k,l}\;:=\partial_{l}u_{k}$ for the gradient of the displacement field $u_{k}$, and ${\ddot{u}}_{j}\;:=\frac{\partial^{2}u_{j}}{\partial t^{2}}$ for the time derivatives. For constant velocity $v_{i}$, this system of equations can be rewritten as ${\hat{C}}_{ijkl}u_{k,il}=0$ with “effective” elastic constants ${\hat{C}}_{ijkl}\;:=\left(C_{ijkl}-\rho v_{i}v_{l}\delta_{jk}\right)$ (see [27]). Upon introducing perpendicular unit vectors ${\vec{m}}_{0}$ and ${\vec{n}}_{0}$, which are normal to the sense vector $\vec{t}$ of the dislocation, i.e., $\vec{t}={\vec{m}}_{0}\times {\vec{n}}_{0}$, the solution takes the form $u_{j,k}\left(r,\phi \right)={\tilde{u}}_{j,k}\left(\phi \right)/r$ where ${\tilde{u}}_{j,k}\left(\phi \right)$ is a function of Burgers vector, $\vec{m}$, $\vec{n}$, and ${\hat{C}}_{ijkl}$ [28] (p. 476):

(3) $$\begin{array}{cc} {\tilde{u}}_{\;j,k} & =\frac{b_{l}}{2\pi }\left\{n_{k}{\left[{\left(nn\right)}^{-1}\left(nm\right)\;\cdot \;S\right]}_{jl}-m_{k}S_{jl}+n_{k}{\left(nn\right)}_{ji}^{-1}K_{il}\right\},\\ S & =-\frac{1}{2\pi }\int_{0}^{2\pi }{\left(nn\right)}^{-1}\left(nm\right)\;d\phi \;,\;\;K=-\frac{1}{2\pi }\int_{0}^{2\pi }\left[\left(mn\right){\left(nn\right)}^{-1}\left(nm\right)-\left(mm\right)\right]d\phi , \end{array}$$

with the shorthand notation ${\left(ab\right)}_{jk}\;:=a_{i}{\hat{C}}_{ijkl}b_{l}$. Variables *r* and ϕ are polar coordinates in the plane spanned by $\vec{m}={\vec{m}}_{0}\left(\vartheta \right)\cos\phi +{\vec{n}}_{0}\sin\phi$ and $\vec{n}={\vec{n}}_{0}\cos\phi -{\vec{m}}_{0}\left(\vartheta \right)\sin\phi$, where ${\vec{n}}_{0}$ is the slip plane normal and ${\vec{m}}_{0}\left(\vartheta \right)$ is perpendicular to ${\vec{n}}_{0}$ and $\vec{t}\left(\vartheta \right)=\frac{1}{b}\left[\vec{b}\cos\vartheta +\vec{b}\times {\vec{n}}_{0}\sin\vartheta \right]$. As such, ${\vec{m}}_{0}\left(\vartheta \right)$ depends on the dislocation character angle ϑ and is parallel to $\vec{v}$. The important feature to note is that ${\tilde{u}}_{j,k}\left(\phi \right)$ includes terms proportional to ${\left(nn\right)}^{-1}$ and hence exhibits divergences whenever det(nn)=0. This happens at certain combinations of polar angle ϕ and critical velocity $|{\vec{v}}_{c}|$. As shown in Ref. [20], critical velocities are typically close to (and sometimes equal to) the lowest shear wave speed associated with the direction of $\vec{\;v}$ in the single crystal. All dislocation displacement gradients computed with the present method are hence restricted to (constant) velocities *v* that are *smaller* than $v_{c}$.

As noted in the previous section, dislocation field $u_{j,k}\left(r,\phi \right)$ (more precisely its Fourier transform $d_{jk}\left(\vec{\;q}\right)$) enters Γ within Equation (2), and thus the drag coefficient *B* depends quadratically on $u_{j,k}$. For simplicity, we presently only consider perfect dislocations; incorporating more realistic models of the dislocation core as well as the effect of partial dislocations into the dislocation drag coefficient are beyond the scope of the present paper and we leave those considerations to future work. For recent advances on the theoretical modeling of dislocation cores (albeit disconnected from phonon wind theory), see [29,30,31,32,33] and references therein.

The slip systems we have considered here are:

(4) $$\begin{array}{ccc} {\vec{b}}^{\mathrm{fcc}}=\frac{b^{\mathrm{fcc}}}{\sqrt{2}}\left(1,1,0\right), & b^{\mathrm{fcc}}=\frac{a}{\sqrt{2}}, & {\vec{n}}_{0}^{\mathrm{fcc}}=\frac{1}{\sqrt{3}}\left(-1,1,-1\right),\\ {\vec{b}}^{\mathrm{bcc}}=\frac{b^{\mathrm{bcc}}}{\sqrt{3}}\left(1,-1,1\right), & b^{\mathrm{bcc}}=\frac{a\sqrt{3}}{2}, & {\vec{n}}_{0}^{\mathrm{bcc}}=\frac{1}{\sqrt{2}}\left(1,1,0\right),\\ {\vec{b}}^{\mathrm{hcp}}=b^{\mathrm{hcp}}\left(-1,0,0\right), & b^{\mathrm{hcp}}=a, & {\vec{n}}_{0}^{\text{hcp-basal}}=\left(0,0,1\right),\\ {\vec{n}}_{0}^{\text{hcp-prismatic}}=\left(0,-1,0\right), & {\vec{n}}_{0}^{\text{hcp-pyramidal}}=\left(0,-a,c\right)/\sqrt{a^{2}+c^{2}} & \end{array}$$

where *a* and *c* are the lattice constants given in Table 1 and Table 2, and $\vec{b}$ and ${\vec{n}}_{0}$ denote the Burgers vector and slip plane normals, respectively (see Refs. [13,20] for details). For the case of close-packed hexagonal (hcp) crystals, we assume the basal plane is normal to the third axis in Cartesian crystal coordinates. The three hcp slip systems we consider, basal, prismatic, and pyramidal slip, share the same Burgers vector but have different slip plane normals. All except for the bcc slip system above lead to expressions that are symmetric with respect to ϑ→−ϑ, and all slip systems are π-periodic.

In Table 1 and Table 2, we list all input data that were used in the computation of the drag coefficient below. For the effective Lamé constants of the polycrystal, we have chosen to use the separate experimental values (where available) listed in those tables rather than analytically averaging over the single crystal values. The only exceptions are Mo and Zr due to lack of experimental data, and because the Voigt and Reuss bounds are very close to each other in those cases. In fact, for SOEC of cubic crystals, analytic averaging would be a viable avenue as well (assuming negligible texturing), but not so much for hcp and other crystals, see [34] and references therein. (The single crystal averages for the Lamé constants of cubic crystals agree well — within a few percent — with the experimental results listed in Table 1, with the exception of Ni whose averaged shear modulus is ∼11% higher than the measured value, and also Au whose averaged λ is ∼12% lower than the measured value.)

### 2.2. The Low Velocity Limit

In the limit of small velocity *v*, small meaning $v\ll c_{T}$ and $v\ll c_{S}$ (where $c_{T}$ is the effective polycrystalline transverse sound speed and $c_{S}$ is the lowest shear wave speed of the single crystal in the direction of $\vec{\;v}$), drag coefficient *B* simplifies to

(5) $$\begin{array}{cc} B & \approx \frac{4\pi }{\hbar }\sum_{s^{\prime },s^{\prime \prime }}\int_{\mathrm{BZ}}\;\frac{d^{3}q^{\prime }}{{\left(2\pi \right)}^{3}}\int_{\mathrm{BZ}}\;\frac{d^{2}q}{{\left(2\pi \right)}^{2}}\;{\left|\Gamma_{s^{\prime }s^{\prime \prime }}\left(\vec{\;q}{\;}^{{\;}^{\prime }},\vec{\;q}{\;}^{{\;}^{\prime }}\;\;-\vec{\;q},\vec{\;q},v=0\right)\right|}^{2}\\ & \;\times {\left(\vec{\;q}\cdot \hat{v}\right)}^{2}\frac{\partial (-n_{q^{\prime }})}{\partial \omega_{q^{\prime }}}\delta \left(\omega_{q^{\prime }}-\omega_{q^{\prime }-q}\right)+C\;v+O\left(v^{2}\right), \end{array}$$

where $\hat{v}$ denotes the unit vector in the direction of $\vec{\;v}$. Explicit numerical calculations for a number of metals show that the first order velocity correction has a *negative* coefficient C<0. To understand why this is the case, we note that the dislocation field itself depends only on the square of its velocity and thus its Taylor expansion around small *v* has no linear term. Furthermore, since $\omega_{q^{\prime }}=c_{s^{\prime }}q^{\prime }$ and Γ scales as $1/\omega_{q^{\prime }}\omega_{q^{\prime \prime }}$, the drag coefficient depends on the sound speeds as $1/c_{s^{\prime }}^{3}c_{s^{\prime \prime }}^{2}$. Since $c_{T}∼c_{L}/2$, the largest contribution to *B* at low velocity *v* is due to the purely transverse branch (where both incoming and outgoing phonons are transverse), as already observed in earlier work [13,14,25]. In this case, it is convenient to introduce a dimensionless integration variable proportional to the ratio $t\propto |\vec{\;q}{\;}^{{\;}^{\prime }}\left|/\right|\vec{\;q}|$. The energy conserving delta function then restricts the integration range of this new variable *t* such that it shrinks with growing dislocation velocity *v* (see [13,14]). This is the dominating effect and the reason for negative *C*.

### 2.3. High Velocity Limit

Our use of an isotropic Debye phonon spectrum introduces the limitation $v<c_{T}$ on our present theory. Nonetheless, *B* does not diverge at $v=c_{T}$: All divergences within *B* are inherited from the poles present in the dislocation field, as pointed out in Section 2.1 above. Indeed, those appear at critical velocities $v_{c}$, which depend on the slip geometry, material constants, as well as the dislocation character ϑ. To determine the highest degree of divergence, we first recall the study done in Ref. [14] in the purely isotropic limit and only for the transverse phonon modes: There it is found that the highest degree of divergence of a dislocation field for pure edge is $1/{\left(1-\beta_{T}^{2}\right)}^{m}$ with $\beta_{T}\;:=v/c_{T}$ and m=1 at polar angle ϕ=0 (or π), whereas the one for pure screw exhibited the milder divergence of m=1/2. Within *B*, where the dislocation field enters quadratically and angles ϕ are integrated over, this leads to initial estimates for the degree of divergence of *B* of m=3/2 for pure edge and m=1/2 for pure screw. However, within the purely transverse branch, the kinematic terms in Γ additionally suppresses the degree of divergence by 1, ultimately leading to $B∼1/{\left(1-\beta_{T}^{2}\right)}^{m}$ as $\beta_{T}\to 1$ with m=1/2 for edge and finite *B* for screw dislocations.

In the more general semi-isotropic case considered here, this latter cancellation cannot occur because now we have divergences at $v_{c}\left(\vartheta \right)$, whereas the kinematic terms in Γ coming from the phonons only know about $c_{T}$ and $c_{L}$. Similarly, the cancellation leading to the milder divergence of the pure screw dislocation in the isotropic limit is indeed special to the strictly isotropic case: For an isotropic screw dislocation, $S\;\cdot \;\vec{\;b}\to \vec{\;0}$, $K\;\cdot \;\vec{\;b}∼\left(0,0,\sqrt{1-\beta_{T}^{2}}\right)$, and ${\left(nn\right)}^{-1}∼1/\left(1-\beta_{T}^{2}\right)$ within Equation (3) yield the milder divergence noted above. Finally, one must also not forget that edge and screw dislocations decouple only in the isotropic limit, but not in general, which is why mixed dislocations cannot be represented as superpositions of edge and screw in “real” crystals.

To sum up: we presently expect the highest degree of divergence of the drag coefficient B(v,ϑ) at $v\to v_{c}\left(\vartheta \right)$ to be $1/{\left(1-v^{2}/v_{c}^{2}\right)}^{m}$ with m=3/2 for arbitrary dislocation characters ϑ. Indeed, this expectation is confirmed by numerical results, where the asymptotic region cannot be well represented by fitting functions with m<3/2.

## 3. Results and Their Effective Functional Form

Based on the analysis of the previous section, the simplest form of a fitting function for the drag coefficient at fixed temperature, pressure, and dislocation character angle which captures its velocity dependence in the small *v* as well as in the asymptotic regime $v\to v_{c}$ is given by

(6) $$\begin{array}{cc} B(\vartheta ) & \approx C_{0}\left(\vartheta \right)-C_{1}\left(\vartheta \right)x+C_{2}\left(\vartheta \right)\left(\frac{1}{{\left(1-x^{2}\right)}^{3/2}}-1\right)\;,\\ x & =\frac{v}{v_{c}\left(\vartheta \right)}=\beta_{T}\frac{c_{T}}{v_{c}\left(\vartheta \right)}. \end{array}$$

**Figure 1:**
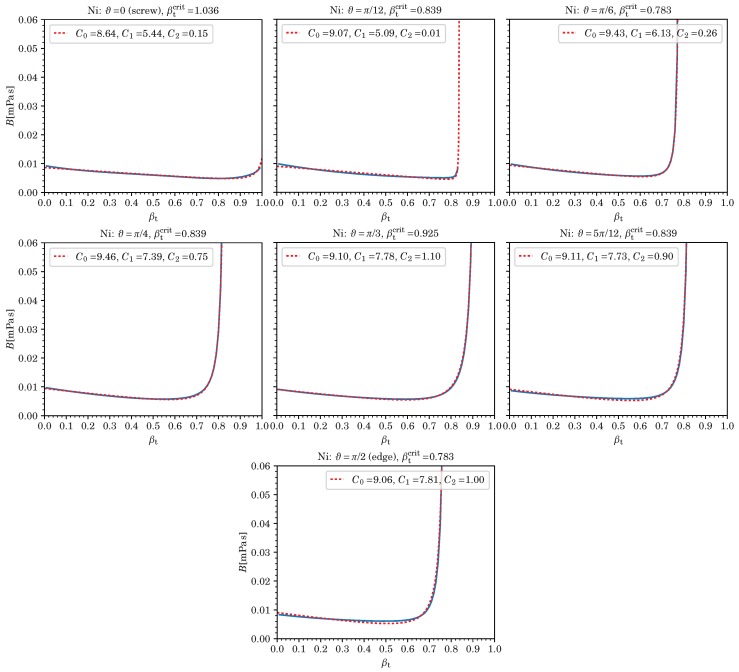
We show the drag coefficient $B(\beta_{T})$ from phonon wind for dislocations in Ni of various character angles ϑ. The dashed lines represent the three-parameter fitting functions with fitting parameters $C_{i}$ in units of μPas, critical velocity $\beta_{t}^{\mathrm{crit}}=v_{c}/c_{T}$, and $\beta_{T}=v/c_{T}$. The solid lines show the results of numerically evaluating *B* according to Equation (1) using the software in Ref. [24], see Ref. [13] for details on the method.

As illustrated in Figure 1 for the example of Ni at room temperature and ambient pressure for a number of dislocation character angles ranging from pure screw (ϑ=0) to pure edge (ϑ=π/2), Equation (6) is perfectly sufficient in some cases. Corresponding fitting parameters (in units of μPas) and critical velocities—all dependent on ϑ—are listed in the figure legends and titles. However, if *B* shows a stronger *v* dependence in the intermediate region, which is the case for a number of metals and slip systems, additional terms are required to improve the fits. Candidates for such additional terms include of course any polynomial $x^{k}$ with k≥2 or subleading divergences (which are always present), e.g., ${\left(1-x^{2}\right)}^{-m}$ with 0<m<3/2 or $\ln(1-x^{2})$. Our goal is to keep *B* simple and the number of fitting parameters small. Empirically, we found that adding only one additional term, ${\left(1-x^{2}\right)}^{-1/2}$, greatly improves the fits in most cases where Equation (6) is insufficient.

Hence, better fits to the drag coefficient from phonon wind for a dislocation of fixed character angle ϑ are achieved using the function

(7) $$\begin{array}{cc} B(\vartheta ) & \approx C_{0}\left(\vartheta \right)-C_{1}\left(\vartheta \right)x+C_{2}\left(\vartheta \right)\left(\frac{1}{\sqrt{1-x^{2}}}-1\right)+C_{3}\left(\vartheta \right)\left(\frac{1}{{\left(1-x^{2}\right)}^{3/2}}-1\right)\;,\\ x & =\frac{v}{v_{c}\left(\vartheta \right)}=\beta_{T}\frac{c_{T}}{v_{c}\left(\vartheta \right)}. \end{array}$$

Once again, it depends on the velocity in ratio to the critical velocity $v_{c}\left(\vartheta \right)$. Note that, since (nn) is a 3×3 matrix, one always has three solutions for det(nn)=0, and each can be represented as $v_{c}\left(\phi \right)$. The branch with the smallest value for $v_{c}$ will lead to a divergence in ${\left(nn\right)}^{-1}$ first. However, that solution need not always lead to a divergent drag coefficient since kinematics restrict the range of polar angle ϕ. This happens for example for pure screw dislocations in fcc metals where *B* diverges at a larger critical velocity $v_{c}$ than the dislocation field itself.

**Figure 2:**
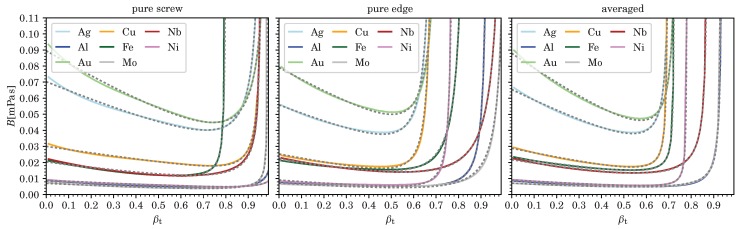
We show the drag coefficient $B(\beta_{T})$ from phonon wind for pure screw and edge dislocations as well as *B* averaged over all character angles ϑ for five fcc and three bcc metals. The dashed lines represent the fitting functions and $\beta_{T}=v/c_{T}$.

**Figure 3:**
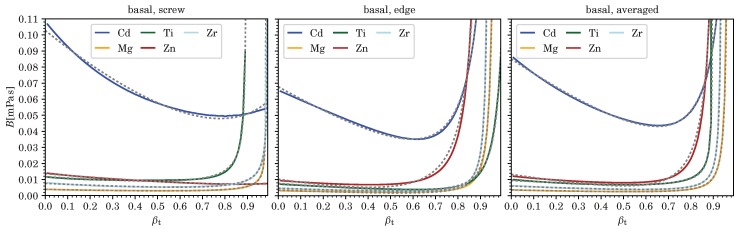
We show the drag coefficient $B(\beta_{T})$ from phonon wind for pure screw and edge dislocations as well as averaged over all character angles ϑ for basal slip of five hcp metals. Dashed lines represent the fitting functions and $\beta_{T}=v/c_{T}$.

**Figure 4:**
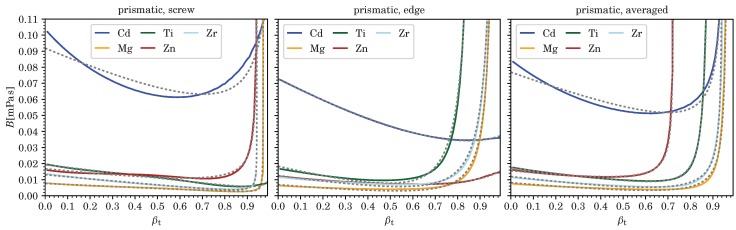
We show the drag coefficient $B(\beta_{T})$ from phonon wind for pure screw and edge dislocations as well as averaged over all character angles ϑ for prismatic slip of five hcp metals. Dashed lines represent the fitting functions and $\beta_{T}=v/c_{T}$.

**Figure 5:**
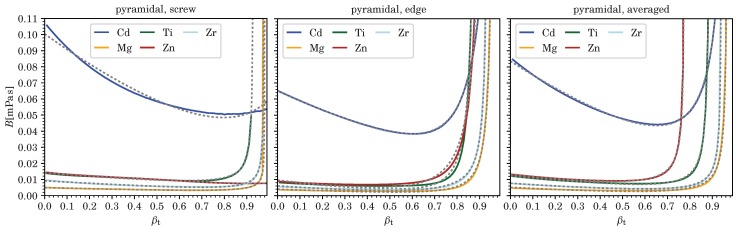
We show the drag coefficient $B(\beta_{T})$ from phonon wind for pure screw and edge dislocations as well as averaged over all character angles ϑ for pyramidal slip of five hcp metals. Dashed lines represent the fitting functions and $\beta_{T}=v/c_{T}$.

According values for $v_{c}$ corresponding to divergences in *B*, as well as the five fitting parameters $C_{i}$ for pure screw and edge dislocations, and for *B* averaged over all dislocation character angles, and each for the various metals computed (at room temperature and ambient pressure), are listed in Table 3, Table 4, Table 5 and Table 6. (Averages were computed as mean values from B(ϑ) for 91 character angles, 0≤ϑ≤π/2 with B(−ϑ)=B(ϑ), for fcc and hcp metals, and from 181 character angles, −π/2<ϑ≤π/2, for bcc metals using [24].) Comparisons of these fits to the numerically computed results for *B* are shown in Figure 2, Figure 3, Figure 4 and Figure 5 as a function of velocity over effective transverse sound speed of the polycrystal, $\beta_{T}=v/c_{T}$. Fits using Equation (7) can of course be derived for any other character angle ϑ. All numerical results presented here can be reproduced with the software in Ref. [24] developed by the present author.

With the exception of Ag, Au, and Cd, most metals shown here have *B* well below 0.04mPas in the low velocity regime, for the most part due to lower values of their transverse sound speeds $c_{T}$ (see Table 3 and Table 4).

Figure 1, Figure 2, Figure 3, Figure 4 and Figure 5 show that B(v,ϑ) at ambient temperature and pressure are well represented by Equation (7) (or even Equation (6)), especially in light of the uncertainty in our current model for *B*, which is hard to quantify. (In our example of nickel, both Equations (6) and (7), yield exactly the same fit for pure edge dislocations, whereas, in the case of screw dislocations, the four-parameter Equation (7) slightly improves an already decent fit by making use of the additional term $1/\sqrt{1-x^{2}}$, thereby changing also the values of the three other fitting parameters.)

For one, we considered only isotropic phonons, whose spectrum deviates from the true one especially in the high frequency regime. Furthermore, we have neglected the dislocation core as well as the separation of dislocations into partials. However, the uncertainties in the experimental (or computational) determination of the TOEC also have a large effect on the accuracy of our present predictions. We also need to stress that the present model is limited to the subsonic regime, i.e., $v<v_{c}\left(\vartheta \right)$ and $v<c_{T}$. Furthermore, only straight dislocations moving at constant velocity were considered, i.e., the effect of acceleration or changes in shape are not (yet) considered. Finally, the stress field required to reach velocities close to $v_{c}$ will likely lead to sizeable temperature and pressure gradients, which would have to be considered in future improvements to *B* as well. A first attempt at incorporating the temperature dependence into *B* is currently in progress [50].

Direct comparison of *B* to experiments is limited to the low velocity regime (low meaning the viscous regime of $\beta_{T}∼0.01$): As (in part) pointed out in Ref. [13], our predictions for $B(\beta_{T}∼0.01)$ agree well with experimental results for Al (ranging from ∼0.005mPas to ∼0.06mPas, cf. [51,52,53]) and Cu (ranging from ∼0.0079 mPas to ∼0.08 mPas, cf. [54,55,56,57,58]. MD simulation results are in the range ∼0.007–0.2 mPas for Al [8,59,60], and ∼0.016–0.022 mPas for Cu [10,61].

Our predictions are lower than experimental results for Fe (∼0.34 mPas for edge and ∼0.661 mPas for screw, cf. [62]) and Zn for both basal slip (0.035 mPas for edge and ∼0.034 mPas for screw, cf. [63]) as well as for pyramidal slip (0.27 mPas for edge and ∼0.16 mPas for screw, cf. [64]).

Our drag coefficient for Mo is lower than the MD-simulation value of ∼0.078 mPas for edge dislocations reported in [65]. Similarly, our drag coefficient for Ni is lower than the MD-simulation results of 0.0321 mPas for edge dislocations reported in [65], and ∼0.015 mPas for edge dislocations reported in [8,12,66], albeit close to the latter.

## Figures and Tables

**Table 1:** List of input data for cubic crystals used in the calculation of the drag coefficient; all elastic constants are given in units of GPa. The references we used to compile these data are: Ref. [35] (Section 12) (lattice parameters *a* and densities ρ), Refs. [36] (p. 10) and [37] (effective Lamé constants of the polycrystal except for Mo), Ref. [35] (Section 12) (single crystal SOEC and Zener anisotropy ratio $A\;:=2c_{44}/\left(c_{11}-c_{12}\right)$), and Refs. [38,39,40,41,42,43] (TOEC). The Lamé constants of Mo (marked with *) are analytical averages of the single crystal SOEC (see, e.g., [34]). The conventions for the single crystal elastic constants are those of Brugger [44].

	Ag${}_{\left(\mathrm{fcc}\right)}$	Al${}_{\left(\mathrm{fcc}\right)}$	Au${}_{\left(\mathrm{fcc}\right)}$	Cu${}_{\left(\mathrm{fcc}\right)}$	Fe${}_{\left(\mathrm{bcc}\right)}$	Mo${}_{\left(\mathrm{bcc}\right)}$	Nb${}_{\left(\mathrm{bcc}\right)}$	Ni${}_{\left(\mathrm{fcc}\right)}$
*a* (Å)	4.09	4.05	4.08	3.61	2.87	3.15	3.30	3.52
ρ (g/ccm)	10.50	2.70	19.30	8.96	7.87	10.20	8.57	8.90
λ (GPa)	83.6	58.1	198.0	105.5	115.5	176.4 *	144.5	126.1
μ (GPa)	30.3	26.1	27.0	48.3	81.6	125.0 *	37.5	76.0
$c_{11}$	123.99	106.75	192.44	168.30	226.00	463.70	246.50	248.10
$c_{12}$	93.67	60.41	162.98	121.20	140.00	157.80	134.50	154.90
$c_{44}$	46.12	28.34	42.00	75.70	116.00	109.20	28.73	124.20
*A*	3.04	1.22	2.85	3.21	2.70	0.71	0.51	2.67
$c_{111}$	−843	−1076	−1729	−1271	−2720	−3557	−2564	−2040
$c_{112}$	−529	−315	−922	−814	−608	−1333	−1140	−1030
$c_{123}$	189	36	−233	−50	−578	−617	−467	−210
$c_{144}$	56	−23	−13	−3	−836	−269	−343	−140
$c_{166}$	−637	−340	−648	−780	−530	−893	−168	−920
$c_{456}$	83	−30	−12	−95	−720	−555	137	−70

**Table 2:** List of input data for hcp crystals used in the calculation of the drag coefficient; all elastic constants are given in units of GPa. The references we used to compile these data are: Ref. [35] (Section 12) (lattice parameters *a*, *c* and densities ρ), Refs. [36] (p. 10) and [37] (effective Lamé constants of the polycrystal except for Zr), Ref. [35] (Section 12) (single crystal SOEC), and Refs. [45,46,47,48,49] (TOEC). The Lamé constants of Zr (marked with *) are analytical averages of the single crystal SOEC (see, e.g. [34]). The conventions for the single crystal elastic constants are those of Brugger [44].

(hcp)	Cd	Mg	Ti	Zn	Zr
*a* (Å)	2.98	3.21	2.95	2.67	3.23
*c* (Å)	5.62	5.21	4.68	4.95	5.15
ρ (g/ccm)	8.69	1.74	4.51	7.13	6.52
λ (GPa)	28.8	24.1	78.5	43.1	71.3 *
μ (GPa)	19.2	17.3	43.8	43.4	36.0 *
$c_{11}$	114.50	59.50	162.40	163.68	143.40
$c_{12}$	39.50	26.12	92.00	36.40	72.80
$c_{44}$	19.85	16.35	46.70	38.79	32.00
$c_{13}$	39.90	21.80	69.00	53.00	65.30
$c_{33}$	50.85	61.55	180.70	63.47	164.80
$c_{111}$	−2060	−663	−1358	−1760	−767
$c_{112}$	−114	−178	−1105	−440	−697
$c_{123}$	−110	−76	−162	−210	37
$c_{144}$	227	−30	−263	−10	37
$c_{113}$	−197	30	17	−270	−96
$c_{133}$	−268	−86	−383	−350	−271
$c_{155}$	−332	−58	117	250	−271
$c_{222}$	−2020	−864	−2306	−2410	−1450
$c_{333}$	−516	−726	−1617	−720	−2154
$c_{344}$	−171	−193	−383	−440	−271

**Table 3:** List of critical velocities $v_{c}$ (m/s), and fitting function coefficients $C_{i}$ (μPa s) for some cubic crystals. The critical velocities are given in units of m/s as well as in ratio to $c_{T}$. The fits are only valid up to $0.99c_{T}$ and do not capture the asymptotic behavior for those metals/dislocations whose critical velocity is larger than this value. Superscripts “e”, “s”, and “av” refer to “edge”, “screw”, and “average”, respectively. Furthermore, $v_{c}^{\mathrm{av}}$ coincides with the smallest critical velocity for all dislocation characters ϑ within the slip system.

	Ag${}_{\left(\mathrm{fcc}\right)}$	Al${}_{\left(\mathrm{fcc}\right)}$	Au${}_{\left(\mathrm{fcc}\right)}$	Cu${}_{\left(\mathrm{fcc}\right)}$	Fe${}_{\left(\mathrm{bcc}\right)}$	Mo${}_{\left(\mathrm{bcc}\right)}$	Nb${}_{\left(\mathrm{bcc}\right)}$	Ni${}_{\left(\mathrm{fcc}\right)}$
$c_{T}$	1699	3109	1183	2322	3220	3501	2092	2922
$v_{c}^{e}/c_{T}$	0.707	0.942	0.739	0.698	0.852	1.033	1.026	0.783
$v_{c}^{e}$	1202	2929	874	1621	2745	3615	2147	2288
$C_{0}^{e}$	55.89	7.84	79.17	25.30	21.79	7.18	22.42	9.06
$C_{1}^{e}$	37.63	6.54	56.08	18.49	17.99	8.25	23.08	7.81
$C_{2}^{e}$	0.00	0.00	0.00	0.00	0.00	0.00	7.70	0.00
$C_{3}^{e}$	4.85	1.25	5.83	2.38	4.18	2.44	3.98	1.00
$v_{c}^{s}/c_{T}$	0.973	1.005	0.996	0.976	0.803	0.987	0.955	1.036
$v_{c}^{s}$	1652	3126	1178	2267	2585	3457	1997	3027
$C_{0}^{s}$	70.26	8.43	89.52	30.02	20.42	7.01	20.56	8.90
$C_{1}^{s}$	54.70	7.28	80.20	20.14	15.96	6.15	18.08	6.81
$C_{2}^{s}$	14.87	2.96	26.88	1.32	5.45	2.76	10.38	2.12
$C_{3}^{s}$	1.38	0.00	0.74	0.92	0.14	0.02	0.00	0.03
$v_{c}^{\mathrm{av}}/c_{T}$	0.707	0.942	0.739	0.698	0.726	0.935	0.875	0.783
$v_{c}^{\mathrm{av}}$	1202	2929	874	1621	2337	3272	1831	2288
$C_{0}^{\mathrm{av}}$	65.50	8.71	88.01	28.91	23.00	7.10	21.77	9.13
$C_{1}^{\mathrm{av}}$	47.73	9.02	69.83	20.25	14.37	6.12	19.28	6.37
$C_{2}^{\mathrm{av}}$	14.79	5.46	19.38	6.13	5.42	5.36	13.12	2.23
$C_{3}^{\mathrm{av}}$	0.32	0.22	0.37	0.14	0.05	0.10	0.09	0.07

**Table 4:** List of critical velocities $v_{c}$ (m/s), and fitting function coefficients $C_{i}$ (μPa s) for basal slip. The critical velocities are given in units of m/s as well as in ratio to $c_{T}$. The fits are only valid up to $0.99c_{T}$ and do not capture the asymptotic behavior for those metals/dislocations whose critical velocity is larger than this value. Superscripts “e”, “s”, and “av” refer to “edge”, “screw”, and “average”, respectively. Furthermore, $v_{c}^{\mathrm{av}}$ coincides with the smallest critical velocity for all dislocation characters ϑ within the basal slip system.

Basal	Cd	Mg	Ti	Zn	Zr
$c_{T}$	1486	3153	3118	2466	2350
$v_{c}^{e}/c_{T}$	1.017	0.972	1.033	0.943	0.943
$v_{c}^{e}$	1511	3065	3219	2326	2215
$C_{0}^{e}$	67.80	3.37	7.59	10.18	4.61
$C_{1}^{e}$	79.08	4.53	10.40	18.71	4.61
$C_{2}^{e}$	0.00	0.00	1.18	0.00	1.25
$C_{3}^{e}$	15.60	1.09	2.05	8.54	0.64
$v_{c}^{s}/c_{T}$	1.398	0.982	0.896	1.211	0.990
$v_{c}^{s}$	2077	3097	2795	2987	2327
$C_{0}^{s}$	102.79	3.88	11.79	13.62	7.60
$C_{1}^{s}$	157.86	2.68	8.21	13.32	6.25
$C_{2}^{s}$	162.12	2.88	8.92	6.80	5.58
$C_{3}^{s}$	0.00	0.00	0.04	0.00	0.01
$v_{c}^{\mathrm{av}}/c_{T}$	1.017	0.972	0.896	0.943	0.943
$v_{c}^{\mathrm{av}}$	1511	3065	2795	2326	2215
$C_{0}^{\mathrm{av}}$	85.14	3.55	9.45	13.28	5.97
$C_{1}^{\mathrm{av}}$	94.67	2.91	6.70	20.08	4.98
$C_{2}^{\mathrm{av}}$	40.73	0.67	5.90	0.00	2.40
$C_{3}^{\mathrm{av}}$	5.22	0.54	0.00	5.40	0.30

**Table 5:** List of critical velocities $v_{c}$ (m/s), and fitting function coefficients $C_{i}$ (μPa s) for prismatic slip. The critical velocities are given in units of m/s as well as in ratio to $c_{T}$. The fits are only valid up to $0.99c_{T}$ and do not capture the asymptotic behavior for those metals/dislocations whose critical velocity is larger than this value. Superscripts “e”, “s”, and “av” refer to “edge”, “screw”, and “average”, respectively. Furthermore, $v_{c}^{\mathrm{av}}$ coincides with the smallest critical velocity for all dislocation characters ϑ within the prismatic slip system.

Prismatic	Cd	Mg	Ti	Zn	Zr
$c_{T}$	1486	3153	3118	2466	2350
$v_{c}^{e}/c_{T}$	1.398	0.982	0.896	1.211	0.990
$v_{c}^{e}$	2077	3097	2795	2987	2327
$C_{0}^{e}$	72.75	6.88	18.34	12.13	12.61
$C_{1}^{e}$	98.25	10.56	28.28	16.52	18.57
$C_{2}^{e}$	81.91	0.00	0.00	18.94	0.00
$C_{3}^{e}$	0.00	2.80	6.99	0.66	4.65
$v_{c}^{s}/c_{T}$	1.017	0.972	1.033	0.945	0.943
$v_{c}^{s}$	1511	3065	3219	2332	2215
$C_{0}^{s}$	91.85	7.65	19.30	16.80	13.08
$C_{1}^{s}$	59.25	6.21	17.41	13.04	12.39
$C_{2}^{s}$	32.17	0.44	0.95	8.37	1.42
$C_{3}^{s}$	0.00	0.01	0.09	0.02	0.00
$v_{c}^{\mathrm{av}}/c_{T}$	0.948	0.972	0.896	0.724	0.943
$v_{c}^{\mathrm{av}}$	1409	3065	2795	1786	2215
$C_{0}^{\mathrm{av}}$	77.00	8.10	17.44	15.65	12.00
$C_{1}^{\mathrm{av}}$	47.08	11.10	16.39	9.88	15.58
$C_{2}^{\mathrm{av}}$	19.87	6.16	0.54	8.73	10.75
$C_{3}^{\mathrm{av}}$	0.00	0.45	1.55	0.00	0.02

**Table 6:** List of critical velocities $v_{c}$ (m/s), and fitting function coefficients $C_{i}$ (μPa s) for pyramidal slip. The critical velocities are given in units of m/s as well as in ratio to $c_{T}$. The fits are only valid up to $0.99c_{T}$ and do not capture the asymptotic behavior for those metals/dislocations whose critical velocity is larger than this value. Superscripts “e”, “s”, and “av” refer to “edge”, “screw”, and “average”, respectively. Furthermore, $v_{c}^{\mathrm{av}}$ coincides with the smallest critical velocity for all dislocation characters ϑ within the pyramidal slip system.

Pyramidal	Cd	Mg	Ti	Zn	Zr
$c_{T}$	1486	3153	3118	2466	2350
$v_{c}^{e}/c_{T}$	1.017	0.972	0.896	0.945	0.943
$v_{c}^{e}$	1511	3065	2795	2332	2215
$C_{0}^{e}$	65.30	3.97	8.92	9.58	6.02
$C_{1}^{e}$	65.48	4.92	11.86	15.05	8.68
$C_{2}^{e}$	5.02	0.00	0.00	0.00	8.38
$C_{3}^{e}$	11.70	1.31	2.45	5.80	0.48
$v_{c}^{s}/c_{T}$	1.278	0.979	0.930	1.132	0.976
$v_{c}^{s}$	1900	3088	2898	2792	2294
$C_{0}^{s}$	100.41	4.94	14.13	14.14	9.17
$C_{1}^{s}$	126.97	3.47	10.47	10.11	7.81
$C_{2}^{s}$	97.54	2.02	6.06	1.65	3.67
$C_{3}^{s}$	0.00	0.00	0.03	0.09	0.01
$v_{c}^{\mathrm{av}}/c_{T}$	0.975	0.972	0.896	0.775	0.943
$v_{c}^{\mathrm{av}}$	1450	3065	2795	1911	2215
$C_{0}^{\mathrm{av}}$	83.61	5.10	12.20	13.34	7.66
$C_{1}^{\mathrm{av}}$	87.21	6.76	11.80	11.41	8.49
$C_{2}^{\mathrm{av}}$	49.38	5.56	5.97	9.78	6.14
$C_{3}^{\mathrm{av}}$	0.83	0.25	0.48	0.00	0.06

## References

[1] Krasnikov V.S., Kuksin A.Yu., Mayer A.E., Yanilkin A.V. (2010). Plastic deformation under high-rate loading: The multiscale approach. Phys. Solid State.

[2] Barton N.R., Bernier J.V., Becker R., Arsenlis A., Cavallo R., Marian J., Rhee M., Park H.S., Remington B.A., Olson R.T. (2011). A multiscale strength model for extreme loading conditions. J. Appl. Phys..

[3] Hansen B.L., Beyerlein I.J., Bronkhorst C.A., Cerreta E.K., Dennis-Koller D. (2013). A dislocation-based multi-rate single crystal plasticity model. Int. J. Plast..

[4] Hunter A., Preston D.L. (2015). Analytic model of the remobilization of pinned glide dislocations from quasi-static to high strain rates. Int. J. Plast..

[5] Borodin E.N., Mayer A.E. (2015). Structural model of mechanical twinning and its application for modeling of the severe plastic deformation of copper rods in Taylor impact tests. Int. J. Plast..

[6] Luscher D.J., Mayeur J.R., Mourad H.M., Hunter A., Kenamond M.A. (2016). Coupling continuum dislocation transport with crystal plasticity for application to shock loading conditions. Int. J. Plast..

[7] Austin R.A. (2018). Elastic precursor wave decay in shock-compressed aluminum over a wide range of temperature. J. Appl. Phys..

[8] Olmsted D.L., Hector L.G. Jr., Curtin W.A., Clifton R.J. (2005). Atomistic simulations of dislocation mobility in Al, Ni and Al/Mg alloys. Mod. Simul. Mater. Sci. Eng..

[9] Marian J., Caro A. (2006). Moving dislocations in disordered alloys: Connecting continuum and discrete models with atomistic simulations. Phys. Rev..

[10] Wang Z.Q., Beyerlein I.J. (2008). Stress orientation and relativistic effects on the separation of moving screw dislocations. Phys. Rev..

[11] Gilbert M.R., Queyreau S., Marian J. (2011). Stress and temperature dependence of screw dislocation mobility in *α*-Fe by molecular dynamics. Phys. Rev..

[12] Daphalapurkar N.P., Wilkerson J.W., Wright T.W., Ramesh K.T. (2014). Kinetics of a fast moving twin boundary in nickel. Acta Mater..

[13] Blaschke D.N. (2019). Velocity dependent dislocation drag from phonon wind and crystal geometry. J. Phys. Chem. Solids.

[14] Blaschke D.N., Mottola E., Preston D.L. (2018). On the Velocity Dependence of the Dislocation Drag Coefficient from Phonon Wind.

[15] Alshits V.I., Indenbom V.L., Lothe J. (1992). The Phonon-Dislocation Interaction and its Role in Dislocation Dragging and Thermal Resistivity. Elastic Strain Fields and Dislocation Mobility.

[16] Rosakis P. (2001). Supersonic Dislocation Kinetics from an Augmented Peierls Model. Phys. Rev. Lett..

[17] Li Q., Shi S.Q. (2002). Dislocation jumping over the sound barrier in tungsten. Appl. Phys. Lett..

[18] Jin Z., Gao H., Gumbsch P. (2008). Energy radiation and limiting speeds of fast moving edge dislocations in tungsten. Phys. Rev..

[19] Ruestes C.J., Bringa E.M., Rudd R.E., Remington B.A., Remington T.P., Meyers M.A. (2015). Probing the character of ultra-fast dislocations. Sci. Rep..

[20] Blaschke D.N., Szajewski B.A. (2018). Line tension of a dislocation moving through an anisotropic crystal. Phil. Mag..

[21] Markenscoff X., Huang S. (2009). The energetics of dislocations accelerating and decelerating through the shear-wave speed barrier. Appl. Phys. Lett..

[22] Pellegrini Y.P. (2010). Dynamic Peierls-Nabarro equations for elastically isotropic crystals. Phys. Rev..

[23] Pellegrini Y.P. (2017). Causal Stroh formalism for uniformly-moving dislocations in anisotropic media: Somigliana dislocations and Mach cones. Wave Motion.

[24] Blaschke D.N. PyDislocDyn. https://github.com/dblaschke-LANL/PyDislocDyn.

[25] Al’shits V.I., Mitlianskij M.D., Kotowski R.K. (1979). The phonon wind as a non-linear mechanism of dislocation dragging. Arch. Mech..

[26] Brailsford A.D. (1972). Anharmonicity Contributions to Dislocation Drag. J. Appl. Phys..

[27] Bacon D.J., Barnett D.M., Scattergood R.O. (1980). Anisotropic continuum theory of lattice defects. Prog. Mater. Sci..

[28] Hirth J.P., Lothe J. (1982). Theory of Dislocations.

[29] Clouet E. (2011). Dislocation core field. I. Modeling in anisotropic linear elasticity theory. Phys. Rev..

[30] Szajewski B.A., Hunter A., Beyerlein I.J. (2017). The core structure and recombination energy of a copper screw dislocation: A Peierls study. Phil. Mag..

[31] Pellegrini Y.P. (2018). Uniformly-moving non-singular dislocations with ellipsoidal core shape in anisotropic media. J. Micromech. Molec. Phys..

[32] Boleininger M., Swinburne T.D., Dudarev S.L. (2018). Atomistic-to-continuum description of edge dislocation core: Unification of the Peierls-Nabarro model with linear elasticity. Phys. Rev. Mater..

[33] Blaschke D.N., Szajewski B.A. (2019).

[34] Blaschke D.N. (2017). Averaging of elastic constants for polycrystals. J. Appl. Phys..

[35] Rumble J.R. (2018). CRC Handbook of Chemistry and Physics.

[36] Hertzberg R.W., Vinci R.P., Hertzberg J.L. (2012). Deformation and Fracture Mechanics of Engineering Materials.

[37] Kaye G.W.C., Laby T.H. Tables of Physical and Chemical Constants. web edition. www.kayelaby.npl.co.uk/.

[38] Thomas J.F. (1968). Third-Order Elastic Constants of Aluminum. Phys. Rev..

[39] Hiki Y., Granato A.V. (1966). Anharmonicity in Noble Metals; Higher Order Elastic Constants. Phys. Rev..

[40] Powell B.E., Skove M.J. (1984). Linear and volume compressibilities and isothermal third-order elastic constants. J. Appl. Phys..

[41] Voronov F.F., Prokhurov V.M., Gromnitskaya E.L., Ilina G.G. (1978). Second- and third-order elastic moduli of a molybdenum single crystal. Phys. Met. Metallogr..

[42] Graham L.J., Nadler H., Chang R. (1968). Third-Order Elastic Constants of Single-Crystal and Polycrystalline Columbium. J. Appl. Phys..

[43] Riley M.W., Skove M.J. (1973). Higher-Order Elastic Constants of Copper and Nickel Whiskers. Phys. Rev..

[44] Brugger K. (1965). Pure Modes for Elastic Waves in Crystals. J. Appl. Phys..

[45] Saunders G.A., Yoğurtçu Y.K. (1986). The effect of hydrostatic and uniaxial pressure on the elastic constants of cadmium. J. Phys. Chem. Solids.

[46] Naimon E.R. (1971). Third-Order Elastic Constants of Magnesium. I. Experimental. Phys. Rev..

[47] Ramji Rao R., Menon C.S. (1973). Lattice Dynamics, Third-Order Elastic Constants, and Thermal Expansion of Titanium. Phys. Rev..

[48] Swartz K.D., Elbaum C. (1970). Third-Order Elastic Constants of Zinc. Phys. Rev..

[49] Singh A., Rathore R.P.S., Agrawal R.M. (1992). Phonons and elastic constants for scandium, zirconium and magnesium. Acta Phys. Hung..

[50] Blaschke D.N., Burakovsky L., Preston D.L. (2019).

[51] Hikata A., Johnson R.A., Elbaum C. (1970). Interaction of Dislocations with Electrons and with Phonons. Phys. Rev..

[52] Gorman J.A., Wood D.S., Vreeland Jr. T. (1969). Mobility of Dislocations in Aluminum. J. Appl. Phys..

[53] Parameswaran V.R., Urabe N., Weertman J. (1972). Dislocation Mobility in Aluminum. J. Appl. Phys..

[54] Suzuki T., Ikushima A., Aoki M. (1964). Acoustic attenuation studies of the frictional force on a fast moving dislocation. Acta Met..

[55] Zaretsky E.B., Kanel G.I. (2013). Response of copper to shock-wave loading at temperatures up to the melting point. J. Appl. Phys..

[56] Stern R.M., Granato A.V. (1962). Overdamped resonance of dislocations in copper. Acta Met..

[57] Greenman W.F., Vreeland Jr. T., Wood D.S. (1967). Dislocation Mobility in Copper. J. Appl. Phys..

[58] Alers G.A., Thompson D.O. (1961). Dislocation Contributions to the Modulus and Damping in Copper at Megacycle Frequencies. J. Appl. Phys..

[59] Yanilkin A.V., Krasnikov V.S., Kuksin A.Yu., Mayer A.E. (2014). Dynamics and kinetics of dislocations in Al and Al-Cu alloy under dynamic loading. Int. J. Plast..

[60] Cho J., Molinari J.F., Anciaux G. (2017). Mobility law of dislocations with several character angles and temperatures in FCC aluminum. Int. J. Plast..

[61] Oren E., Yahel E., Makov G. (2017). Dislocation kinematics: A molecular dynamics study in Cu. Mod. Simul. Mater. Sci. Eng..

[62] Urabe N., Weertman J. (1975). Dislocation mobility in potassium and iron single crystals. Mater. Sci. Eng..

[63] Pope D.P., Vreeland T. (1969). Mobility of basal dislocations in zinc. Phil. Mag..

[64] Jassby K.M., Vreeland T. (1977). Investigation of pyramidal edge and screw dislocation mobility in zinc by a compressional stress pulse technique. Mater. Sci. Eng..

[65] Weinberger C.R. (2010). Dislocation drag at the nanoscale. Acta Mater..

[66] Bitzek E., Gumbsch P. (2004). Atomistic study of drag, surface and inertial effects on edge dislocations in face-centered cubic metals. Mater. Sci. Eng..

